# A feasibility study on the use of cranial nerve non-invasive neuromodulation to improve affected arm function in people in the chronic stage of a stroke

**DOI:** 10.1186/s12883-025-04213-5

**Published:** 2025-05-16

**Authors:** Maureen Ahiatsi, Guillaume Léonard, Eléonor Riesco, Marie-Claude Girard, Marie-Hélène Milot

**Affiliations:** 1 Research Centre on Aging, Centre intégré universitaire de santé et de services sociaux de l’Estrie – Centre hospitalier universitaire de Sherbrooke (CIUSSS de l’Estrie - CHUS), 1036 Rue Belvédère Sud, Sherbrooke, Québec J1H4C4 Canada; 2 Faculty of Medicine and Health Sciences, Université de Sherbrooke, 3001, 12e Avenue Nord, Sherbrooke, Québec J1H5N4 Canada; 3 Faculty of Physical Activity Sciences, Université de Sherbrooke, 2500, boulevard de l’Université, Sherbrooke, Québec J1K2R1 Canada

**Keywords:** Stroke, Motor function, Upper limb, Strength training program, Cranial nerve non-invasive neuromodulation

## Abstract

**Background:**

Chronic stroke survivors are often left with residual arm muscle weakness impeding arm function, daily life activities and quality of life. Exercise is one of the main post-stroke interventions to improve arm function, with cranial nerve non-invasive neuromodulation (CN-NINM) emerging as a potentially interesting complementary therapy to enhance its benefits. Only one study has evaluated the impact of CN-NINM combined with a lower-limb training program on improved balance in subacute stroke survivors. The aim of this study was to assess the feasibility and explore the effects on motor function of an arm strengthening program combined with CN-NINM in chronic stroke survivors (> 6 months).

**Methods:**

Twelve (12) participants (69 ± 11 years) took part in this feasibility study. Recruitment and drop-out rates, number of people who elected not to participate, adherence and adverse events were collected to assess feasibility. The effects of CN-NINM + exercise on motor function were evaluated by changes in arm motor function, measured using the Fugl-Meyer Assessment (FMA), and functional performance, evaluated through the Wolf Motor Function Test (WMFT), following a 4-week arm strengthening program (60 min, 3 sessions/week) combined with CN-NINM (tongue stimulation, 20 min at a comfortable intensity). Descriptive and non-parametric statistics (Wilcoxon signed-ranks test) were used to describe feasibility data and explore CN-NINM effects.

**Results:**

Feasibility was confirmed with a recruitment rate of 1.3 person/month, no dropout, a 100% adherence rate, and no serious adverse events. A significant gain in FMA (*p* = *0.003*) with a trend for WMFT (*P = 0.11*) were noted post-intervention.

**Conclusion:**

This study suggests that CN-NINM combined with an arm strengthening program is feasible and may improve arm function in chronic stroke survivors. Further research is needed to validate the results.

**Trial registration:**

This clinical trial was registered on ClinicalTrials.gov (NCT05370274) on April 27, 2022.

**Supplementary Information:**

The online version contains supplementary material available at 10.1186/s12883-025-04213-5.

## Background

Stroke is the third most common cause of disability and the second most common cause of death, worldwide [1, 2]. More than 100 million survivors are coping with a variety of stroke-related impairments, such as residual upper limb (UL) weakness [2]. Indeed, over 65% of stroke survivors do experience residual UL muscle weakness, once discharged from rehabilitation, that greatly impacts their daily activities, social participation and quality of life [3–6].

North-American and European Stroke Organisations agree that exercise is one of the best ways to reduce the negative impact of post-stroke residual UL weakness and promote motor recovery [7, 8]. These recommendations are supported by the evidence that exercise increases UL motor function, muscle strength, and motor cortex excitability [9–12], all translating in an increased use of UL in daily tasks. In post-stroke rehabilitation, clinicians usually favour functional training over strength training programs [13–15], based on the premise that strength training programs overexcite muscle pathways, thereby increasing spasticity [16]. In recent years, more and more studies have challenged this idea, and strength training programs are gaining increased popularity in stroke rehabilitation, demonstrating ample benefits over other types of exercise [17, 18]. These benefits can be seen, not only in the development of power, hypertrophy and muscular strength, where a relationship between muscle strength and function in stroke survivors has been demonstrated [18, 19], but also in the generation of neural and structural adaptations [16, 20]. These results align with a recent study, demonstrating that an individualized upper limb (UL) strength training program, tailored to the recovery capacity of each chronic stroke survivor, enhances both motor and functional capabilities across all individuals, irrespective of stroke severity [21–23]. Moreover, recognizing the importance of intensity in achieving treatment benefits and promoting recovery post-stroke [5], strength training programs are generally easier to monitor in this aspect compared to functional training, as there are guidelines available for prescribing intensity in strength training programs [24].

Non-invasive brain neurostimulation (NIBS) techniques are increasingly used as a complementary therapy to optimize the benefits of exercise, and to support motor recovery and neuroplasticity [25–28]. Among NIBS, transcranial direct current stimulation (tDCS) has garnered the most research to date and is crucial for post-stroke functional rehabilitation [26]. Despite the benefits of tDCS on neuroplasticity and motor recovery [29–35], a wide range in response to tDCS is observed, with over 50% of people not responding as expected [25]. The reasons may be the absence of optimal tDCS application parameters, the presence of inter-individual anatomical variability, as well as electrical current shunting through the skull bones [25, 36].

To get over these constraints, a novel NIBS in stroke, called cranial nerve non-invasive neuromodulation (CN-NINM), is making its debut as an adjunct therapy [37]. CN-NINM is based on the principle of facilitating mechanisms of structural and functional neuroplasticity [37–42]. The CN-NINM device can thus bypass the disadvantage of tDCS since it is directly applied to a person’s tongue. The tongue is an interesting and promising target for stimulation, as it possesses many advantageous features that promote the transmission of electrical signals to the brain, such as its high density of nerve fibres, a controlled environment with constant pH, and a low excitability threshold [37, 43]. These signals are delivered by electrical sequences targeting sensory fibres located around 300–400 μm below the surface of the tongue, with the electrical impulses crossing multiple synaptic connections and creating a continuous neuronal flow [43]. Thus, CN-NINM generates a flow of impulses that depolarizes cranial nerves, namely the lingual branch of the trigeminal nerve (CN Vc), responsible for tactile sensations, and the facial nerve (CN VII) [37, 44–47] as well as glossopharyngeal (CN IX), vagus (CN X) and hypoglossal (CN XII) nerves [41, 47]. The resulting neural impulses are transmitted to the corresponding nuclei in the brainstem [37] and then travel to the motor cortex to induce targeted neuroplastic changes when combined with rehabilitation treatments [46]. It is also hypothesized that the stimulation may interact with adjacent vestibular nuclei, activating circuits involved in movement coordination, balance, breathing and consciousness [37]. When CN-NINM is combined with various therapies, it leads to increased motor cortex activity [48, 49] and enhanced functional performance – such as walking [43, 44, 50] and balance [43, 44, 48, 50], in several types of neurological diseases [43, 48]. In stroke, studies on the evaluation of the effects of CN-NINM on motor recovery are scarce. One study has evaluated the effects of CN-NINM when combined with a 2-week lower-limb training program on balance and gait in stroke survivors in the subacute stage [50]. In this study, participants were divided in two groups: a control group receiving a 2-week intensive gait and balance training program (2 × 90-minute sessions per day) and a usual rehabilitation program, and an experimental group receiving the same training programs in combination with CN-NINM (20 min of stimulation at level 5–8 on the controller, no specifications for stimulation frequency). The results demonstrated a significant improvement in balance in the experimental group, as compared to the control group, as assessed by the Mini-Best test score (*p* = 0,032) [50], but no change in gait performance was noted. Similarly, in a 13-month training combined to CN-NINM, a case study by Danilov et al. has shown a reduced fall risk as well as a 48% and 30% improvement in gait (Dynamic Gait Index) and mobility (Timed-up-and-go), respectively, in a 80-year-old woman, 4 years post-stroke [51]. Although promising, CN-NINM has not been studied to improve UL function, despite the negative impact of UL impairment on post-stroke functional performance in chronic stroke survivors.

The aim of this study was to assess the feasibility and explore the effect of CN-NINM combined with a UL strength training program, on affected UL motor function and functional performance, in individuals at the chronic stage of a stroke.

## Materials and methods

### Design and participants

Before describing the participants and the study design, it should be noted that stroke participants in this study took part in another nested study, exploring the immediate effect of a single application of CN-NINM on experimental pain, before starting the current protocol. The results of the effect of CN-NINM on pain will be presented in a companion paper. The pain protocol, which induced pain of short duration using a thermode on participants’ forearm, was conducted on the week before the UL strength training program. To ensure that the pain protocol did not impact the UL motor function protocol, pain scores were measured on a 0–10 visual analogue scale (VAS) both after the removal of the thermode and before the initiation of the UL motor function protocol. The pain scores taken at both time points confirmed that none of the participants experienced residual pain (mean VAS = 0 for all participants).

This study took place at the Research Centre on Aging in Sherbrooke (Québec, Canada). Because CN-NINM studies are scarce, especially following a stroke, sample size was determined based on Julious’ recommendations on pilot studies [52]. Twelve (12) individuals were included in the study and followed these inclusion criteria: (1) be aged ≥ 18 years; (2) have had a single supratentorial stroke; (3) be in the chronic stage of a stroke (> 6 months); (4) presenting at least minimal motor recovery in the affected UL (Fugl-Meyer Stroke Assessment [FMA] score at least 20/66) [53] and (5) having completed all rehabilitation treatments. Participants were excluded if they had: (1) significant spasticity at the affected UL (score > 3 on the modified Ashworth scale) [54]; (2) a major sensory deficit at the affected UL (a score < 6 on the vibration threshold assessment) [55]; (3) presence of hemineglect (Line Cancellation test score ≥ ± 0.083) [56]; (4) apraxia (Alexander test score > 2.5) [57]; (5) presence of a neurological disorder other than stroke-related; (6) orthopaedic problems in the affected UL (e.g., fracture, sprain); (7) cognitive impairment (Mini-Cog score < 2/5); and (8) any contraindication to CN-NINM (e.g., metal implants, active cancer, epilepsy, pacemaker, mouth problems, mouth piercings, pregnancy) [43]. These inclusion and exclusion criteria ensured that the participants understood the instructions and were able to take part in the training program. Prior to study enrolment, all participants signed a written informed consent form and the experimental protocol for this study was approved by the CIUSSS de l’Estrie-CHUS ethics committee. This study was conducted in accordance with the Declaration of Helsinki and the Tri-Council Policy Statement: Ethical Conduct for Research Involving Humans (TCPS 2). This clinical trial was registered on ClinicalTrials.gov (NCT05370274).

### Feasibility outcomes

Following Thabane et al.’s recommendations on key aspects of pilot studies [58], the feasibility of this study was measured by the recruitment rate (number of individuals who were recruited out of the number of individuals who agreed to participate), adherence to the intervention (number of training sessions completed out of the total number expected), drop-outs and number of people who elected not to participate, as well as their reasons for not participating. Finally, at the end of each CN-NINM session, participants completed a home-developed questionnaire to collect information on symptoms that may be related to CN-NINM to record adverse events (see Supplementary file 1). Participants were also asked to indicate whether they had noticed any adverse effects in-between CN-NINM sessions.

### Clinical assessment

UL pain was evaluated at baseline to ensure that no participant had pain that could affect the UL training protocol. Stroke participants rated their pain at rest using a visual analogue scale (VAS) ranging from 0 (no pain) to 10 (worst pain imaginable).

Before and after the intervention, participants underwent a clinical assessment of their affected UL, performed by a trained assessor. The main clinical outcomes related to UL function and functional performance were FMA and Wolf Motor Function Test (WMFT), respectively. The UL FMA portion assessed movement, coordination, and reflexes with a maximum score of 66 reflecting normal motor recovery [53]. The WMFT assessed functional performance of the UL via 15 functional tasks and 2 strength-based tasks. Each task of the WMFT was assessed by a timed score with a maximum time of 120 s. Performance on each task was also scored on a 6-point scale (0 = does not attempt to perform the task with the UL to 5 = the UL participates; his movement appears normal). These tools have good validity and reliability with people with a stroke [59, 60].

Other secondary clinical outcomes included (1) the Motor Activity Log (MAL) assessing participant-reported quantity and quality of use of the UL for 14 daily activities (5 = normal) [61]; (2) grip strength of the affected hand, measured with the JAMAR^®^ dynamometer (average of three trials in kg) and (3) manual dexterity, assessed by the Box and Block test (number of blocks that can be moved in 60 s) [62]. These tools have good validity and reliability in people with a stroke [61, 62].

### Strength training program and CN-NINM

The strength training program parameters followed the American Stroke Association’s recommendations for post-stroke exercise prescription [24]. This training program was chosen over functional training to ensure, as mentioned above, an adequate monitoring of training intensity, a crucial training characteristic following a stroke [18, 19, 23]. Participants were trained 3 times a week for 4 weeks. Each training session consisted of 3 series of 10 repetitions for each movement with a 2-minute rest between series. Each training session lasted 60 min. The targeted muscle groups were wrist extensors, elbow and shoulder flexors and grip muscles, as they are all involved in the functional performance of the UL [63, 64]. Participants trained the different muscle groups by lifting free weights. The maximum load that a person could lift 10 times (10RM) for each muscle group was used to estimate the 1RM, using the Brzycki protocol [65]. Training started at 50% of 1RM and progressed by 10% each week, i.e., 60% of 1RM at week 2, 70% of 1RM at week 3 and 80% of 1RM at week 4. The grip muscles underwent the same gradation of intensity application, but with the JAMAR^®^ dynamometer (Lafayette, IN, USA).

CN-NINM was applied to the participants’ tongue during the first 20 min of each training session (12 sessions), using a portable stimulator (Cthulhu Shield, USA) equipped with 18 electrodes. The CN-NINM produced high-frequency pulses (50 µsec to 150 Hz) [37, 50]. Our choice of this frequency was based on studies having used this stimulation parameter in participants showing similar neurological impairments as stroke individuals [37, 43, 66]. Participants held the CN-NINM in place by pressing their tongue upwards and regulated the intensity of the stimulus to a comfortable level of sensation [43], comparable to the sensation in the mouth of a soft drink [46]. These stimulation characteristics are known to be safe for people with a neurological impairment [43].

### Statistical analyses

Feasibility outcome measures were compiled and summarised in terms of the number of participants who completed the intervention, dropped out and reported CN-NINM-related adverse events, as well as the average percentages of training sessions completed by all participants. Sociodemographic data were analysed using means, standard deviations, medians, and ranges. Wilcoxon signed ranks tests were also used to explore the effect of CN-NINM combined to UL training on changes in clinical outcome measures. The significance level was 0.05 and the statistical analyses were performed using IBM SPSS 25.

## Results

### Baseline sociodemographic and clinical characteristics of participants

The final sample consisted of 12 participants; see Table 1 for baseline characteristics. With a mean FMA score of 47 ± 18, all participants showed sufficient UL motor recovery to undergo the intervention.

**Table 1:** Baseline clinical and demographic characteristics of the participants

	Number (%)	Mean (SD)	Median (IQR)	Range
**Age (years)**		69 (11)	72 (15)	49–82
**Sex**				
Female	2 (17)			
Male	10 (83)			
**Hand dominance**				
Right	11 (92)			
Left	1 (8)			
**Time since stroke (months)**		80 (73)	56 (82)	6–248
**Stroke type**				
Ischemic	9 (75)			
Haemorrhagic	3 (25)			
**Side of stroke**				
Right	5 (42)			
Left	7 (58)			
**Modified Ashworth Scale (/4)**				
Shoulder extensors (normal = 0)		0.4 (0.5)		
Elbow flexors (normal = 0)		0.5 (0.8)		
Wrist flexors (normal = 0)		0.3 (0.5)		
Finger flexors (normal = 0)		0.3 (0.5)		
**Pain intensity on the VAS (/10)**		0 (0)	0 (0)	0–0
**Fugl-Meyer Stroke Assessment score (/66)**		47 (18)	57 (38)	21–65

SD, Standard deviation; %, Frequence; IQR, Interquartile range, VAS; visual analogue scale

### Feasibility

**Fig. 1:**
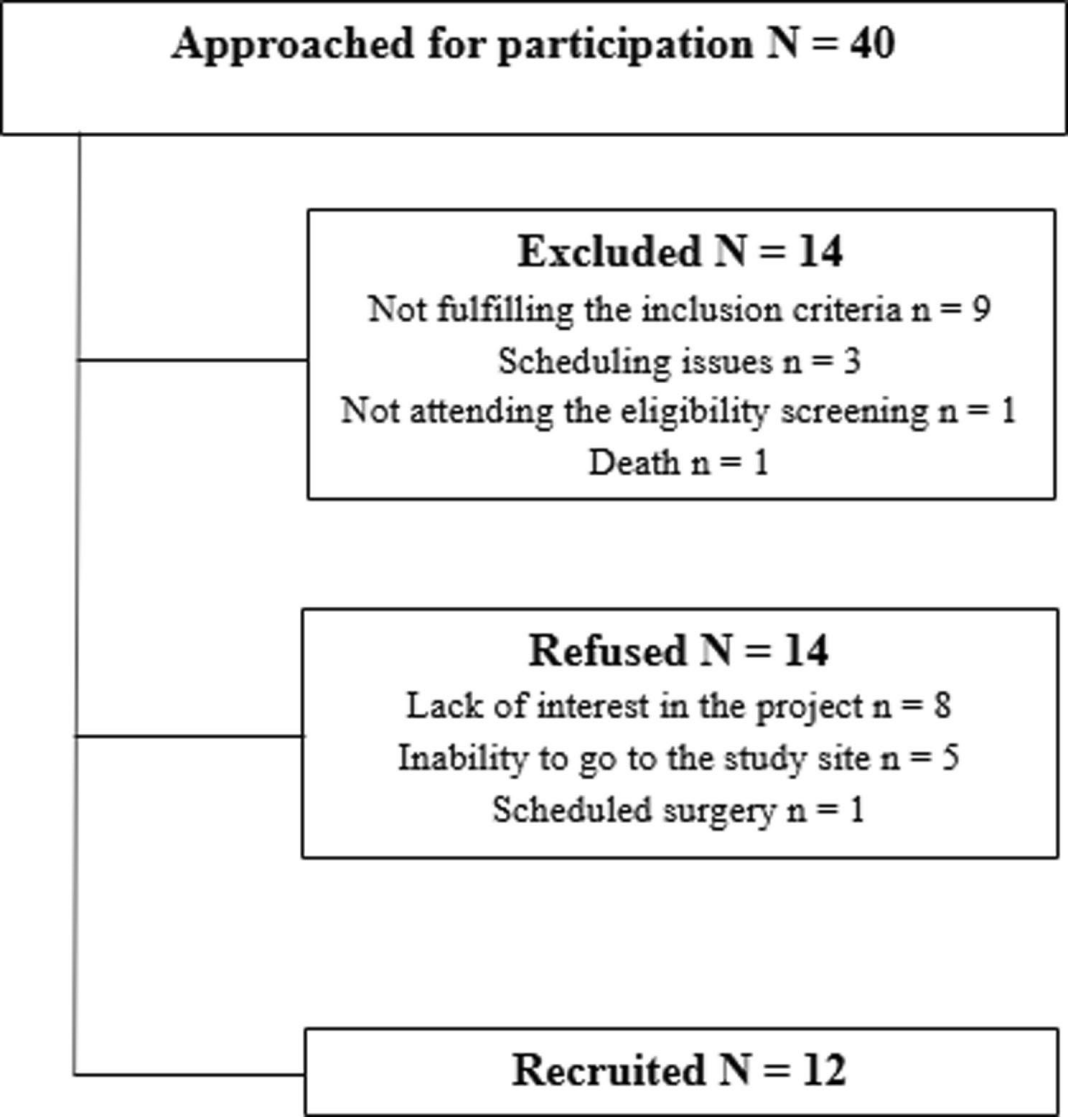
Study recruitment flow diagram

Out of the 40 people who were contacted over a 9-month period, 14 were excluded for the following reasons: not fulfilling the inclusion criteria (*n* = 9), unable to meet the study schedule (*n* = 3), not showing up at the eligibility screening visit (*n* = 1), and death (*n* = 1). For the 14 people who elected not to take part in the project, their reasons were lack of interest in the project (*n* = 8), inability to commute to the study site (*n* = 5), and a scheduled surgery (*n* = 1). Twelve (12) people were thus eligible and willing to participate in the study (see Fig. 1). The recruitment rate was 1.3 people per month, and the 12 participants recruited fully completed all 12 training sessions, following the prescribed protocol (100% adherence). No serious adverse effects were reported by participants, only 7 out of 12 participants reported slight tingling as an CN-NINM related adverse effect, which disappeared quickly (about 10 min post-stimulation). Moreover, none of the participants reported any problems related to the strength training program (e.g., inability to lift the weights or perform the planned repetitions).

### Clinical changes

#### Primary outcome measures

For the FMA, a statistically significant change (*P* = *0.003*) between pre- and post-intervention was observed. For the WMFT, a trend towards an improvement in the score and the time to completion was noted (Score, *P* = 0.07; Time to completion, *P* = 0.09; Weight, *P* = 0.11; see Table 2).

#### Secondary outcome measures

A statistically significant change was noted in the quality and the quantity of use of the affected UL with the MAL test, as reported by the participants (*P = 0.05* for amount of use and *P* = 0.01 for quality of use). Both the Box and Block test and the grip strength test showed no change between pre- and post-intervention (see Table 2).

**Table 2:** Changes in clinical outcomes between pre- and post-CN-NINM paired with UL strength training

Clinical outcomes	Pre	Post	*P*-value^a^
**FMA** (normal = 66)	47 ± 18	51 ± 18	0.003
**WMFT**			
Score (normal = 5)	3.1 ± 1.5	3.3 ± 1.5	0.07
Time to completion (s)	35.7 ± 43.1	30.1 ± 36.9	0.09
Weight (maximum = 20 lbs)	13 ± 6	14 ± 6	0.11
**MAL**			
Amount of use (normal = 5)	2.9 ± 1.5	3.1 ± 1.6	0.05
Quality of use (normal = 5)	2.9 ± 1.4	3.3 ± 1.6	0.01
**Box and Block test**			
Affected side	19 ± 17	20 ± 17	0.23
**Grip strength (kg)**			
Affected side	19 ± 12	20 ± 14	0.53

Note: ^a^Comparison Pre VS Post using Wilcoxon signed ranks test

Abbreviations: FMA, Fugl-Meyer Stroke Assessment; WMFT, Wolf Motor Function test; MAL, Motor Activity Log

## Discussion

This pioneered study investigated the feasibility of using CN-NINM combined with a 4-week strength training program of the affected UL in adults in the chronic phase (≥ 6 months) of a stroke, as well as explored the effect of CN-NINM on UL function and performance. The results showed that it was feasible to use the proposed protocol (CN-NINM + UL training program) in chronic stroke survivors. Significant changes in the FMA and MAL scores were observed following the intervention.

By looking at our flowchart, we can note that equal numbers of people elected not to take part in the project as those who chose to do so. For people who could not participate, reasons were mainly related to commuting, especially for those living in more remote areas, and not related to the protocol *per se.* Thus, we believe that the proposed protocol (CN-NINM combined with a 4-week UL training program) is feasible in adults with chronic stroke, supporting our first hypothesis. Our recruitment rate was 1.3 person/month. Since the study by Galea et al. [50] does not report any recruitment rate, it is difficult to compare our results. A study by Wildenberg et al. evaluated CN-NINM in 9 participants presenting chronic balance problems secondary to various diagnoses and 9 controls, who were recruited over 16 months [67]. Their recruitment rate was 1.1 person/month, relatively close to that of our study. Also, CN-NINM was positively used by all our participants during the 12 training sessions, supporting the intervention’s acceptability in our sample.

No training-related adverse effect was reported by participants that were trained at intensity levels recommended by the American Stroke Association guidelines for post-stroke exercise prescription [24]. Also, no serious CN-NINM-related adverse effects were reported by participants, reinforcing the safety of using this device in stroke, and for the 7 participants that reported minor CN-NINM-related adverse effects, the tingling sensation was transient and did not interfere with the continuation of the intervention sessions. In all, our results corroborate previous studies showcasing the safety and applicability of CN-NINM across diverse neurological conditions, including stroke [37, 46, 50, 66, 68].

Our second hypothesis stated that CN-NINM combined with a 4-week UL strength training program, as proposed in our protocol, would improve motor function in the affected UL of participants. We are aware that we cannot directly attribute the observed improvement in FMA and MAL to CN-NINM alone, but our results support the findings of the study by Galea et al. [50] in stroke, as well as other studies in other neurological populations [43, 48]. Our results also suggest that the combination of our training protocol with CN-NINM stimulation parameters appears suitable and sufficient to enable greater motor recovery. The changes observed in the clinical variables FMA and MAL seem to suggest that our intervention (CN-NINM + UL strength training program) might have modulated structures in the brainstem, corroborating previous studies that have demonstrated the effect of CN-NINM on several brain regions and consequently on brain function [41]. Using a new fMRI signal processing method to yield high-resolution images, Danilov et al. demonstrated that the impulses generated by the CN-NINM stimulated cranial nerves. The impulse then travelled to targeted areas in the brainstem and cerebellum to induce plastic changes [47]. We could hypothesize that a similar mechanism of action might have occurred with our sample following the intervention, but the lack of a control group and brain evaluation assessment do not allow us to draw any conclusions at this time. As CN-NINM is an innovative approach, the precise nature and extent of CN-NINM-induced neuroplastic changes still need further evaluation and must therefore be the subject of future research.

It should also be highlighted that CN-NINM stimulation parameters (intensity, frequency, and pulse width) are highly variable across studies. CN-NINM application can vary from 1 to 3 times a week, with treatment duration ranging from 2 weeks to 7 months and sessions lasting between 20 and 90 min, using either high-frequency pulse (150 pulses/second) or low-frequency pulse (0.08 pulses/second) [37, 43, 48, 68]. Future studies are needed to define the optimal stimulation parameters to maximize CN-NINM’s add-on effects.

### Study limitations

Since this study is a feasibility study with a limited number of participants, results should be interpreted with great care. Thus, a larger sample is needed to confirm and strengthen our preliminary observations. Moreover, our non-probabilistic convenience sampling is subject to selection bias, since only people who wanted to take part in the project did so. Having precise inclusion criteria allowed us to control and maximize the internal validity of our study, but it affected the generalizability of our results to a wider stroke population. The absence of a control group is also a main limitation, as we cannot clearly establish that the results we observed are attributable to CN-NINM per se. For future research, adding a control group would enable rigorous determination of the specific effect of CN-NINM on motor function. Given also that the search for effects was not the main objective of the current feasibility study, our results allow us to conclude only on effects after the four weeks of intervention. In future RCT studies, incorporating a long-term follow-up evaluation would allow to see whether the short-term effects persist over time. It is also important to mention that some of the variables studied – such as adverse effects and MAL – are self-reported measures, which may have generated a desirability bias.

## Conclusion

The present study is the first one to document the feasibility of using CN-NINM combined with a 4-week UL strength training program and to explore the effect of this intervention on motor function of the affected UL in adults in the chronic stage of a stroke. This study provided strong evidence for the feasibility and safety of using repetitive CN-NINM (12 sessions, 3 times a week), with a 100% adherence rate, and no serious CN-NINM-related adverse effects. Thus, the protocol, as proposed in our study, is feasible for use in a future RCT. Also, the intervention translated into improvement in UL motor function and UL quantity/quality of use, suggesting that the protocol might be effective to promote greater recovery in the affected UL of chronic stroke survivors. Further and larger-scale studies are still needed to determine the optimal CN-NINM stimulation parameters, and to better understand CN-NINM induced neuroplastic changes.

## Electronic supplementary material

Below is the link to the electronic supplementary material.

Supplementary Material 1

## Data Availability

The datasets used and/or analysed during the current study are available from the corresponding author on reasonable request.

## Abbreviations

CN: Cranial nerve
CN-NINM: Cranial nerve non-invasive neuromodulation
FMA: Fugl-Meyer Assessment
MAL: Motor activity log
NIBS: Non-invasive brain stimulation
SD: Standard deviation
tDCS: Transcranial direct current stimulation
UL: Upper limb
WMFT: Wolf motor function test
