# Supplementary material for: A feasibility study on the use of cranial nerve non-invasive neuromodulation to improve affected arm function in people in the chronic stage of a stroke
Source: BMC Neurol. 2025 May 16;25:208. doi: 10.1186/s12883-025-04213-5 (PMC12082858; doi:10.1186/s12883-025-04213-5)
Supplement: Supplementary file 1 — Supplementary Material 1 [file 12883_2025_4213_MOESM1_ESM.pdf]

Post-training CN-NINM questionnaire

Have you experienced one of the following symptoms or side effects?	Intensity of symptom<br>1- None<br>2- Light<br>3- Moderate<br>4- Severe	If present, is it related to CN-NINM?<br>1- No<br>2- Not closely related<br>3- Possibly<br>4- Probably<br>5- Definitely	Notes
Headache			
Neck pain			
Tingle			
Itching			
Burning sensation			
Redness of the tongue			
Other (specify)			
